# The relationship and heterogeneity of family participation and social participation among older adults: from an intersectionality perspective

**DOI:** 10.1186/s12877-024-05545-6

**Published:** 2024-11-15

**Authors:** Yan Liu

**Affiliations:** School of Ethnology and Sociology, Minzu University of China, 27 Zhongguancun South Aveue, Beijing, 100081 China

**Keywords:** Family participation, Social participation, Intersectionality, Inequality

## Abstract

Participation in late life has been studied as a component of active aging. To effectively promote late-life participation, the study explored the relationship and heterogeneity between two forms of participation among older adults—familial participation and societal participation. This paper utilizes data from the 2020 China Longitudinal Aging Society Survey (CLASS) to examine the relationship between familial participation and societal participation among older Chinese adults. Linear regression results indicate that familial participation can facilitate societal participation among older adults. The MAIHDA model results suggest variations in familial and societal participation among older adults in different social positions. Specifically, older women with higher socioeconomic status and better health have higher levels of familial and societal participation, whereas older men with lower socioeconomic status and health have the lowest levels of participation. Socioeconomic status is the most significant factor contributing to participation disparities among different groups, and older adults with disadvantaged health status experience a compounding effect of multiple disadvantages. The research findings hold significant implications for formulating policies aimed at enhancing the participation of marginalized older adults, ultimately contributing to the realization of active aging.

## Introduction

In the context of an aging population, it is necessary to encourage the active participation of older adults. China is at a critical stage of this demographic shift. According to the Seventh National Population Census, there are 191 million people aged 65 and above, accounting for 13.5% of the total population. In response to the increasingly severe challenges of an aging population, China has made it a priority to actively respond to demographic changes and has introduced targeted policies. A key concept emerging from these efforts is "active aging," which emphasizes that older adults should have the right to fully participate in society [1]. This entails providing equal and accessible opportunities for them to engage in various activities that improve their quality of life and emotional well-being.

An important aspect of participation for older adults is family participation. As China’s population and family structures evolve, many families now consist of three generations—grandparents, parents, and grandchildren [2]. This shift, along with the introduction of the "three-child" policy, has increased the role of grandparents in childcare and household duties. Many young parents, struggling to balance work and family responsibilities, rely on older family members for support. Consequently, family participation has become an integral part of older adults' lives, particularly after retirement, complementing their social activities. This dual involvement in both family and social participation highlights the increasingly diverse ways in which older adults contribute to their families and communities [3].

However, there are notable differences in both family and social participation across individuals, and it is essential to identify those with lower levels of participation to develop more effective, targeted interventions. Understanding the relationship between family and social participation, as well as the variations within this relationship, is a key step toward enhancing overall social participation among older adults.

One major limitation of existing research is that comprehensive studies exploring the broader relationship between family and social participation are still lacking. Although research has shown that participation in one area can influence involvement in other areas, [4–6] most empirical studies have remained narrow in scope. They primarily focus on the relationship between caregiving within family participation and activities like volunteering or physical exercise as forms of social participation [7–9]. Therefore, the research aims to investigate whether family participation influences social participation among older adults.

A second limitation of previous studies is that existing studies on the factors influencing social participation is their reliance on isolated, "singular" research designs that typically consider only one demographic characteristic or the interaction between two. These approaches overlook the complexity of social identities, failing to account for the diversity and unequal status of marginalized groups that arise from the intersection of multiple identities. For older adults occupying different social positions and possessing varying levels of resources, their family and social participation also differ. Thus, the author adopts an intersectional perspective to explore the heterogeneity in the relationship between family and social participation across different social positions.

In the study, the author proposes two research questions: Question 1: What is the relationship between family participation and social participation? Question 2: Is there heterogeneity in the relationship between family participation and social participation for older adults occupying different social positions?

## Literature review

### Relationship between social participation and family participation

Although there is no universally agreed definition of social participation, and debate remains on whether family activities should be included, the study follows Levasseur et al.'s perspective, the author define social participation as activities where individuals interact with others in society or the community [10]. Social participation is commonly understood as involvement in interpersonal interactions outside the home, including social, leisure, community activities, and work [11]. On the other hand, we define family participation as interpersonal interactions within the family, including caregiving and household chores [12].

Some scholars have found that participation at the family level can restrict older adults' social participation. According to the 2008 China Time Use Survey conducted by the National Bureau of Statistics, the time Chinese older adults spend caring for young children is longer than the time spent caring for adult family members, providing support externally, participating in community services, and engaging in social activities. After retiring, some older adults are mainly responsible for doing housework and looking after children at home. These things take up most of the older adults' time, making it difficult for them to find time to exercise and participate in outside activities [7]. Indeed, in China, approximately one-third of older adults are involved in both caregiving for parents and grandchildren, as well as social participation activities [13]. In Beijing, urban older adults primarily focus on caring for grandchildren, while rural elderly tend to engage more in household chores [14]. Previous research has highlighted the negative impact of caregiving responsibilities within family participation on older adults, suggesting that it may lead grandparents to retire early and reduce or even abandon social participation activities [15, 16]. Intergenerational caregiving, to some extent, hinders older adults’ decisions regarding participation in exercise. Older adults without intergenerational caregiving responsibilities are 1.22 times more likely to participate in exercise than are those with care responsibilities. Older adults’ participation in exercise is limited by care responsibilities. Compared to older adults who often feel that their children demand too much help and support, those without similar care responsibilities have a 72% greater likelihood of participating in exercise [6].

However, some scholars have found that participation at the family level can promote older adults’ social participation. Relevant empirical research has also shown that the types and amount of time spent on family care as part of family participation are beneficial for participating in social activities. Hoyert and Seltzer, using data from the National Survey of Families, analyzed the social activities of adult female caregivers caring for spouses, children or parents. Overall, they found that caregivers participated in more organizations than non-caregivers did [17]. In addition, Schmidt et al. reported positive correlations between forms of social participation, such as volunteer services, educational activities, religious and leisure activities, and decisions regarding the provision of informal care [18].

Therefore, there is no consistent conclusion regarding the relationship between family participation and social participation. Moreover, existing research lacks a holistic perspective on the relationship between older adults’ family participation and social participation. Based on the above review, the author proposes the first research question: Does older adults’ family participation promote or inhibit social participation?

### Explanations for the relationship between family participation and social participation

The theoretical framework on older adults’ resources developed by Arber and Ginn provides a basis for analyzing older adults’ family participation and social participation. This theory proposes the concept of “interlocking resources,” which include material resources, health resources, and care resources. There is a reciprocal relationship between these three types of resources, and changes in them jointly influence individuals’ ability to participate in activities. The theory holds that individuals’ total resource endowment is limited. Older adults need to maintain a balance between family participation and social participation according to their own resource conditions. That is, when a certain type of resource is reduced, the importance of other resources increases. Moreover, this highlights the differential effects of different resources on participation. This provides a useful perspective for understanding the heterogeneous relationships between different forms of participation [19].

Based on Arber and Ginn’s theoretical framework on older adults' resources, the author views social participation as a form of activity. When older adults have relatively abundant resources across material resources, health resources, and care resources, this is most conducive to their social participation. Specifically, in terms of material resources, research shows that a lack of financial resources restricts older adults’ social participation [20]. The impact of self-rated health may be greater than the impact of social participation on health, indicating that health is an important factor driving older adults’ social participation [21, 22]. For care resources, family participation can provide older adults with information regarding opportunities for social participation. Taken together, these three types of resources jointly influence older adults’ motivations and behaviors regarding social participation.

Merely having the resources for participation is insufficient to motivate older adults to engage in social participation. They must also have intrinsic motivations or a willingness to participate. Two main perspectives exist regarding the formation of individual participation motivations. The first is the social psychological perspective, which holds that individuals’ values, beliefs and socialization processes affect their participation in the public sphere. These are considered cultural capital [23]. The second is the behavioral perspective, which emphasizes individuals’ cost‒benefit analyses based on the utility maximization of accumulated social network resources. These network resources can be considered social capital [24]. These two perspectives provide theoretical bases for analyzing individuals’ participation motivations.

Therefore, given the simultaneous presence of resources and willingness, participating in one type of activity can actually enhance the likelihood that individuals participate in other social activities [9]. The main underlying mechanism is that participation in one activity expands individuals’ social networks and relationships. These new social ties provide more opportunities for individuals to participate in social activities in other domains [25]. That is, the social capital accumulated through participating in one activity generates spillover effects on other activities. The information, resources and relationship networks individuals obtain through one activity facilitate their participation across more social roles, thus achieving positive interactions between different types of activity participation. This theory provides possible explanatory pathways for exploring interaction effects between participation across different activities. Based on this discussion, the author proposes the first research hypothesis: Family participation promotes social participation.

### The relationship between social participation and family participation from the perspective of intersectionality

We will explore the relationship between family and social participation among older adults from an intersectional perspective, focusing on how different social positions shape this dynamic. Intersectionality theory emerged in the 1980s and 1990s through the work of Black feminist scholars in the United States, who examined the multiple marginalizations faced by Black women. This theory critiques the limitations of analyzing social categories like gender or race in isolation, emphasizing how the interaction of multiple social identities influences individual experiences. Its core idea is to highlight how various advantages and disadvantages intersect, stressing the need to understand the interaction between different identities rather than viewing marginalization through a single lens [26–28].

Intersectionality provides a crucial framework for analyzing how multiple identities shape social participation. Previous research on the factors influencing social participation has often focused on singular demographic characteristics, such as being male, having low socioeconomic status, being poor health are associated with lower level of social participation [29–32]. These findings suggest that intersectionality offers a deeper analysis of the complexities surrounding participation among older adults, reflecting broader social structures [33].

Traditionally, intersectional analysis has been applied in qualitative research [34–36]. However, in recent years, scholars have begun using existing social identity categories for quantitative analysis [37]. One significant quantitative method for exploring intersectionality is the Multilevel Analysis of Individual Heterogeneity and Discriminatory Accuracy (MAIHDA). This method uses two-level multilevel models in which individuals at level 1 are nested within social strata at level 2, based on combinations of dimensions of social position like gender, race, and socioeconomic status. This approach helps analyze inequalities by looking at both average outcomes and variations within and between these strata, which has several important advantages over conventional, fixed effects approaches for analyzing intersectional data [38]. Such as scalability, model parsimony, small sample size, and interpretability of results [39].

Previous research has not sufficiently explained how overlapping social identities affect social participation, limiting the understanding of participation disparities and the development of targeted policies. By applying an intersectional perspective, the author aims to uncover the diverse participation patterns among older adults from different social positions. This approach builds on previous research that focused on single dimensions by considering how multiple social identities—such as gender, socioeconomic status, and health-interact to influence participation. Based on this, the author proposes the second research hypothesis: older women with higher socioeconomic status and better health are more likely to exhibit a stronger positive correlation between family and social participation. This is because their intersecting advantages in gender, economic, and health status increase their likelihood of participation.

## Data, measures and methods

### Data

The data come from the 2020 China Longitudinal Aging Social Survey (CLASS) [40]. This is a large-scale longitudinal social survey with a nationally representative sample conducted by the National Survey Research Center at Renmin University of China. The survey aimed to explore the social problems and challenges faced by older adults in the aging process and to assess the effectiveness of public policies in improving quality of life. To achieve these goals, CLASS selected individuals aged 60 years and older through multistage probability sampling. Counties (including counties, cities, and districts) were selected as primary sampling units, while villages/resident committees were selected as secondary sampling units. The survey covered 476 villages/resident committees across 30 provinces/autonomous regions/municipalities. Appropriate weights were used to account for the effect of cluster sampling. The data were collected through door-to-door surveys by well-trained student interviewers. After deleting samples with missing values, the final sample size was 10,625.

### Variable measurement

Social Participation: Social participation was measured across five aspects: political participation, social and educational activities, personal entertainment, volunteer work, and physical exercise. Political participation was measured by asking whether the respondent had voted in elections for the local residents/villagers committee in the past three years. Participation was coded as 1, and nonparticipation was coded as 0. Participation in social and educational activities and personal entertainment over the past year was measured by asking about activities such as religious gatherings, attending a university/training course for seniors, watching TV/listening to the radio/reading/reading the newspaper/listening to opera, singing/playing an instrument, playing mahjong/chess/cards, and square dancing. Participation in each activity was coded as 1, and nonparticipation was coded as 0. Volunteer participation was measured by asking whether the respondent had engaged in the past year in environmental sanitation protection or volunteer services requiring professional skills (such as medical consultation and cultural and science promotion). Participation in either was coded as 1, and nonparticipation in both was coded as 0. The frequency of physical exercise participation was also measured. Never exercising was coded as 0, and any participation was coded as 1. In total, the social participation score for each activity ranges from 0 to 10, with higher scores indicating higher levels of participation.

Family Participation: Three aspects of family participation were measured: caring for parents, caring for grandchildren, and helping married children perform housework. Caring for parents was measured by asking how much time the older adult spent per week on average over the past month caring for their parents (and spouse’s parents). A response of 0 h was coded as 0, and any other response was coded as 1. Caring for grandchildren was measured by asking whether the respondent had ever helped care for the children of each of their children, with yes coded as 1 and no coded as 0. Helping married children with housework was measured by asking about the past 12 months how often the respondent helped their married children with housework, with “almost never” coded as 0 and “almost every day” to “a few times a year” coded as 1. Finally, family participation was categorized as no participation, coded as 0; participation in one of the aspects, coded as 1; and participation in two or more aspects, coded as 2.

Health Status: Measurement of health status included physical health and mental health. To comprehensively measure health status, the operationalization included self-rated health, depression, and physical health functioning. Self-rated health was measured by asking respondents to evaluate their current health status. Responses of “very healthy” and “quite healthy” were coded as 0 to indicate good health, “fair” was coded as 1, and “quite unhealthy” and “very unhealthy” were coded as 2 to indicate poor health. Higher scores thus indicate poorer self-rated health. Depression was measured using the 9-item Chinese version of the CESD scale. Scores from 1 to 9 were coded as 0 indicating no depression, scores from 10 to 17 were coded as 1 indicating depressive tendencies, and scores from 17 to 27 were coded as 2 indicating a high risk of depression. Hence, higher scores on this measure indicate greater depression risk. Physical health functioning was measured using the 10-item Instrumental Activities of Daily Living (IADL) scale. A score of 0 indicates normal functioning and was coded as 0. Scores from 1 to 5 were coded as 1, indicating functional impairment; scores from 6 to 10 were coded as 2, indicating significant functional impairment. A composite health status index was constructed using latent class analysis (LCA), based on the individual health measures described above, including self-rated health, depression, and physical health functioning. Model fit indices showed that dividing respondents into two latent health classes provided the best fit (1 class: AIC = 31,656.4, BIC = 31,678.39; 2 classes: AIC = 30,829.31, BIC = 30,880.7; 3 classes: AIC = 30,829.3, BIC = 30,880.9). Respondents could thus largely be divided into two groups – healthy and unhealthy.

Socioeconomic Status: Following measurement approaches used in prior research [41], the operationalization of socioeconomic status included education level, income, and household registration status. In the study, household registration was divided into two categories: 1) agricultural hukou and 2) nonagricultural hukou. Education level was categorized into four groups based on the respondents’ highest level attained: 1) illiterate, 2) elementary school, 3) middle school, and 4) high school and above. Income was divided into four groups according to personal annual income: 1) low income (less than 2300 RMB); 2) lower-middle income (2300–5000 RMB); 3) upper-middle income (5000–10,000 RMB); and 4) high income (above 10,000 RMB). Through latent class analysis (LCA), a composite socioeconomic status index was constructed based on the individual measures above. Model fit comparisons showed that a 2-class model showed the best fit based on the education, income and household registration variables selected (1 class: AIC = 29,699.19, BIC = 29,713.87; 2 classes: AIC = 28,800.79, BIC = 28,822.82; 3 classes: AIC = 28,808.79, BIC = 28,860.18). Respondents could thus largely be divided into two socioeconomic status groups—high and low.

Control Variables: Based on findings from existing research, the control variables in the study included sex, age, income, marital status, and health status.

### Methods of analysis

To address research question 1, the author fitted a linear regression model and used propensity score matching to test the robustness of the results.

For research question 2, the author used the MAIHDA model [39] to estimate the interaction effects and predicted effects at each intersectional stratum. Each individual is assigned to a specific intersectional stratum, which is combined of social categories for a group of individuals who share intersecting social identities. In this case, the following social categories were considered: gender (male vs. female), health status (unhealthy vs. healthy), socioeconomic status (high vs. low), and family participation (none vs. one type vs. two or more types). This means that each older adult is assigned to one of the strata (i.e., 2 × 2 × 2 × 3 = 24), where each stratum represents a unique combination of gender, health status, socioeconomic status, and family participation. The author began by estimating the intersectional effects using two models: a basic intersectional model and an intersectional interaction model. From each model, the author obtained measures of general and specific intersectionality effects. General intersectionality was assessed using the variance partition coefficient (VPC) and proportional change in variance (PCV), which tells us how much of the differences between individuals within stratum are explained by additive effects, with the unexplained portion attributable to interaction effects. Specific intersectionality was assessed using the predicted mean values of each stratum and the strata-level residuals. The analysis utilized a Bayesian multilevel framework. Specifically, Bayesian Markov chain Monte Carlo (MCMC) estimation methods were used by fitting models in Stan via the R package “brms,” an interface for fitting Bayesian generalized (non)linear multivariate multilevel models [42]. The author specified non or very weakly informative priors for all parameters and ran analyses with a chain of 5,000 iterations in the warm-up phase and a total length of 10,000 iterations.

## Results

### Descriptive statistics results

Table 1 provides the descriptive statistical results of the main variables of the intersection analysis. There were 5530 men and 5295 women. The mean social participation of women (2.70) was slightly greater than that of men (2.66); in terms of health status, 6338 were unhealthy and 4287 were healthy. The average social participation of healthy elderly people (2.86) was greater than that of unhealthy elderly people (2.56). Finally, in terms of socioeconomic status, there were 4963 elderly people with high socioeconomic status and 5662 elderly people with low socioeconomic status. Among them, the social participation level of elderly people with higher socioeconomic status (3.09) is much greater than that of elderly people with lower socioeconomic status (2.33). The descriptive results revealed the distribution of socioeconomic status according to demographic characteristics. However, to explore the role of social location formed by the overlap of different demographic characteristics on the social participation of elderly people, further analysis is needed.

**Table 1:** Descriptive analysis results

Descriptive analysis of main variables in intersectional analysis		
Variable Name	Sample Size	Mean Social Participation Score (Std Dev)
Gender		
Men	5330	2.66(1.67)
Women	5295	2.70(1.80)
Health Status		
Unhealthy	6338	2.56(1.78)
Healthy	4287	2.86(1.64)
Socioeconomic Status		
High	4963	3.09(1.84)
Low	5662	2.33(1.56)
Family Participation		
None	3669	2.23(1.42)
Middle	4487	2.63(1.65)
High	2469	3.44(2.02)
Overall	10,625	2.68(1.74)
Description of the main variables of linear regression		
Variable name	Sample size	Mean (standard deviation)
Social participation	10,625	1.86(0.95)
Family involvement	10,625	1.35(1.49)
Gender	10,625	0.50(0.50)
Age	10,625	71.46(6.49)
Married	10,625	0.77(0.42)
Education level	10,625	1.50(0.75)
Hukou	10,625	0.53(0.50)
Income	4205	2.44(1.15)
Self-rated health	10,609	3.37(0.90)
Body functions	10,625	19.40(3.79)
Community facilities	10,625	1.63(1.51)

Table 1 provides the descriptive statistical results of the main variables of the linear regression. The level of social participation is 1.86, which shows that the social participation status of elderly people is low. Similarly, the level of family participation is 1.351, which shows that the average level of family participation of elderly people is not high. In addition, the sex distribution of all the samples was relatively balanced. The average age was 70.46 years (SD = 6.491). A total of 77% of the elderly people had spouses, and the number of elderly people with agricultural household registrations and nonagricultural household registrations was equal.

### Regression analysis results

The linear regression results support the first research hypothesis that family participation promotes social participation. Appendix Table 6 reports the linear regression results. Regardless of whether the control variables were considered, family participation always had a positive effect on older adults’ social participation (*p* < 0.01). After adding control variables, for older adults engaging in family participation, the likelihood of participating in social activities increased by 10.6%. Moreover, considering potential endogeneity issues with some control variables, substitution of variable measurements and use of propensity score matching (PSM) methods (see Tables 2 and 3) showed the results to be robust.

**Table 2:** Robustness test of linear regression analysis results of family participation and social participation

Methods	Sample	Have Family Participation = (1)	No Family Participation = (2)	ATT = (1)-2)	SE	t-Value
Proximity matching (K = 1)	Unmatched	1.79	1.70	0.08	0.02	5.16***
	matched	1.78	1.72	0.06	0.01	4.33***
Kernel matching	Unmatched	1.79	1.70	0.08	0.02	5.16***
	matched	1.78	1.74	0.04	0.02	2.20***

^***^p < .01, ^**^p < 0.05, ^*^p < 0.1

**Table 3:** Robustness test of linear regression analysis results of family participation and social participation

Methods	Sample	Ps R^2^	LR chi^2^	*p* > chi^2^	Mean Bias	Med Bias
Proximity matching (K = 1)	Unmatched	0.035	224.97	0.000	11.2	10.1
	matched	0.002	10.35	0.582	2.8	3.3
Kernel matching	Unmatched	0.035	224.97	0.000	11.2	10.1
	matched	0.001	3.99	0.828	1.4	1.3

The replacement time measurements remain significant. In the measurement of the family part, the measurement of caring for the elderly was replaced from whether to care for the elderly individual to "the time you spent taking care of this child's children in the past 12 months". Family participation was remeasured, and then a linear regression model was conducted. Fitting was used to determine that the impact of family participation (0.096, *p* = 0.095) on social participation was still positive.

To correct for possible biases in the variable selection process, the author used the propensity score matching (PSM) method for testing. Table 2 shows that after using k-nearest neighbor (KNN) and kernel matching (kernel matching) methods for robustness testing, the results are as follows. The average treatment effect (ATT) of the treated is consistent with previous empirical results. Specifically, there is a significant positive correlation between family participation and social participation among older Chinese adults. According to the balance test results in Table 3, after matching for most variables, the standardized errors are less than 10%, eliminating the imbalance between control variables and meeting the requirements of PSM. After PSM, the robustness of the results was further verified.

### MAIHDA intersectional analysis

Table 4 shows that being male, being unhealthy, having a lower socioeconomic status, and lacking family participation are associated with significantly lower levels of social participation. To test the extent to which intersections of social identity categories help explain inequality in social participation, the author used the intersectional measure of proportional change in variance (PCV). The variance in social participation across intersectional layers decreased from VPC = 13.14% in the empty model to VPC*adj* = 2.17% in the main effects model. This means that the interaction effects of social identity categories were smaller than the additive effects. The PCV showed that 85.33% of between-layer differences in social participation could be explained by additive main effects, while 14.67% was attributable to interaction effects. In previous research applying MAIHDA approaches in health-related contexts, PCVs ranged from -37.93% to 99.85%, with a median of 92.92%. Thus, the PCV from the example is of comparable magnitude to the PCVs reported in prior studies.

**Table 4:** Parameter estimates of multi-level models of social participation levels

	**Null model [95% CI]**	**Main model [95% CI]**
**Fixed effects**		
Intercept	0.37[0.27, 0.50]	0.12[-0.06, 0.07]
Gender		
Female (reference)		
Male		-0.01[-0.06, 0.06]
Health status		
Unhealthy		-0.07[-0.13, -0.01]
Health (reference)		
Socioeconomic status		
High (reference)		
Low		-0.20[-0.26, -0.13]
Family involvement		
None (reference)		
Category 1 and above		0.23[0.17, 0.28]
**Measures of variance**		
Variance level 2: Intersectional strata	0.1335	0.0196
Variance level 2: Individuals	0.8828	0.8830
**VPC**	**13.14%**	**2.17%**
**PCV**		**85.33%**

*95% CI* 95% credible intervals, *VPC* variance partition coefficient, *PCV* proportional change in the between-strata variance

**Fig. 1:**
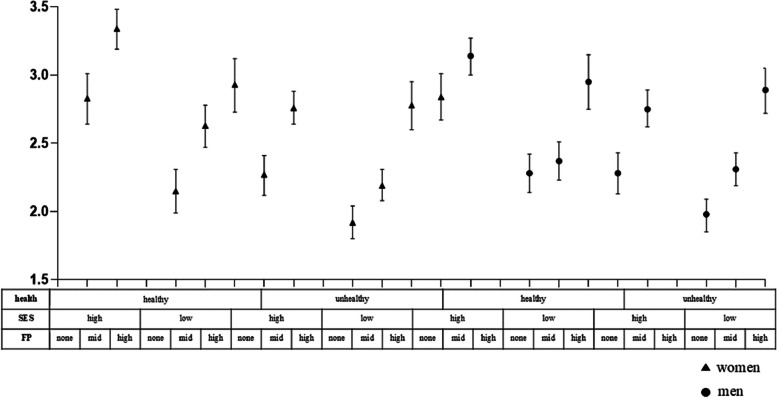
Stratum-specific (intersectional) predicted mean values for social participation level (and 95% credible interval) based on the intersectional interaction model

**Fig. 2:**
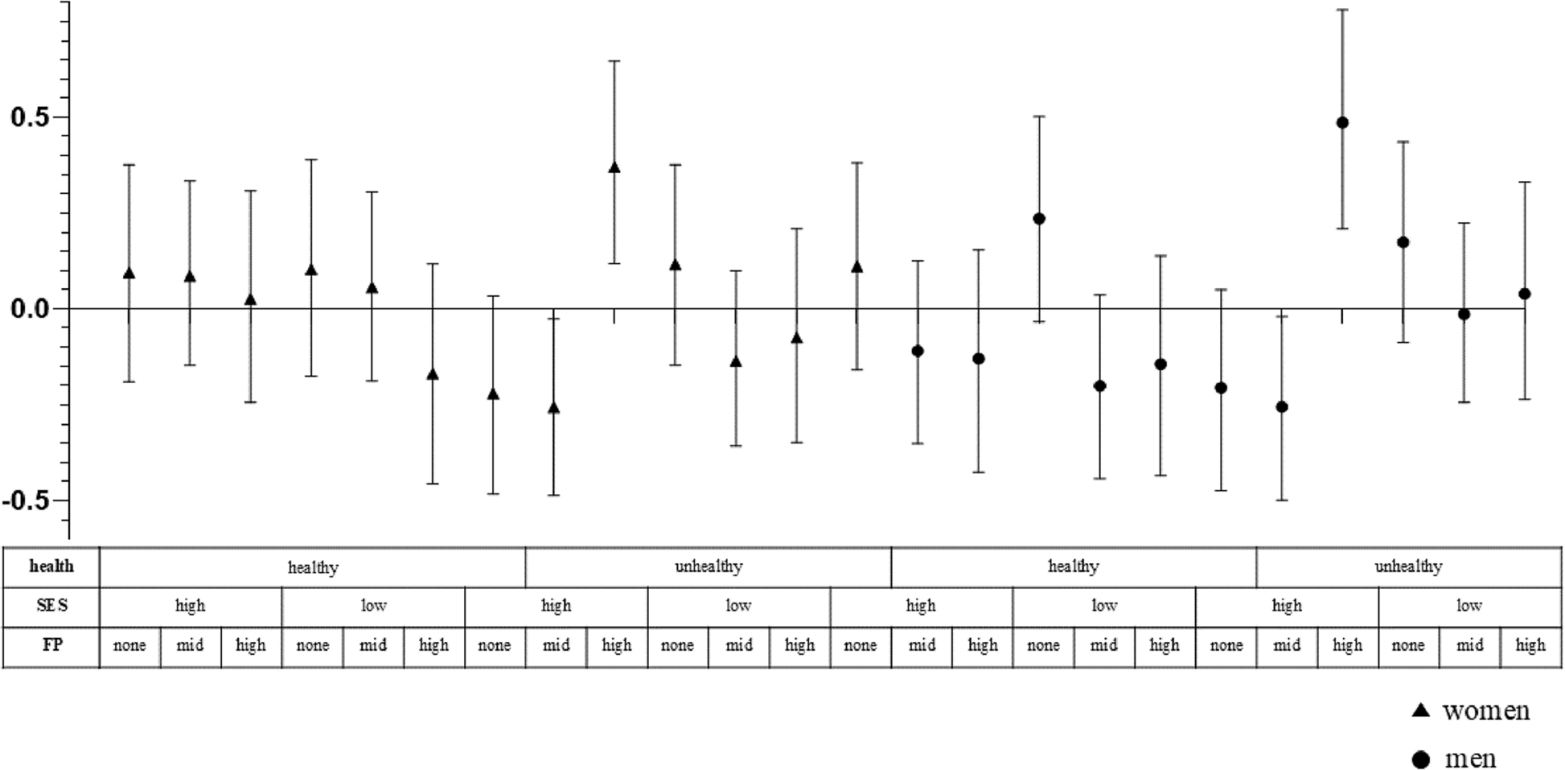
Stratum-level residuals for social participation based on the intersectional interaction model

Specifically, Fig. 1 and Table 5 show the results for social participation levels at each intersection layer. The layer with the highest social participation was healthy women of high socioeconomic status with high family participation (0.630, 95% CI [0.488,0.780], while the layer with the lowest participation was unhealthy men of low socioeconomic status without family participation (-0.504, 95% CI [-0.641, 0.358]). To examine whether social participation inequality within specific intersectional layers was more or less pronounced due to interaction effects, the author calculated interaction effects for each layer (see Fig. 2 and Table 5), considered to be specific measures of intersectionality. The author found four significant intersectional layer interaction effects. Among the unhealthy groups, men and women with high socioeconomic status and family participation had stratified residuals of -0.149 (95% CI [-0.289, 0.013]) and 0.277 (95% CI [0.124, 0.441]), respectively, indicating that social participation was influenced by interaction effects stemming from their intersectional layers.

**Table 5:** Sample sizes for all 24 cross-layers calculated using MCMC estimation method, mean level of social participation (95% confidence interval), expected level of social participation (based on fixed effects only), predicted level of social participation (based on fixed effects only) and random effects) and random effects (95% confidence interval)

						**Null model [95% CI]**	**Main model [95% CI]**		
ID	N	gender	health	SES	FP	Predicted sp level	Expected sp level	Predicted sp level	Random effects
1	256	women	healthy	high	none	0.095[-0.015, 0.208]	0.028 [-0.117, 0.174]	0.085 [-0.023, 0.194]	0.057 [-0.103, 0.224]
2	460	women	healthy	high	middle	0.381[0.295, 0.466]	0.329 [0.205, 0.457]	0.380 [0.299, 0.464]	0.051 [-0.085, 0.195]
3	297	women	healthy	high	high	0.637[0.530, 0.744]	0.630 [0.488, 0.780]	0.648 [0.545, 0.747]	0.017 [-0.145, 0.178]
4	359	women	healthy	low	none	-0.291[-0.385, -0.196]	-0.367 [-0.510, -0.223]	-0.306 [-0.400, -0.211]	0.061 [-0.097, 0.217]
5	381	women	healthy	low	middle	-0.026[-0.120, 0.069]	-0.066 [-0.186, 0.059]	-0.032 [-0.118, 0.054]	0.034 [-0.109, 0.168]
6	217	women	healthy	low	high	0.118[-0.001, 0.243]	0.235 [0.093, 0.379]	0.142 [0.024, 0.257]	-0.093 [-0.26, 0.066]
7	441	women	unhealthy	high	none	-0.248[-0.336, -0.161]	-0.109 [-0.252, 0.039]	-0.239 [-0.321, -0.156]	-0.13 [-0.289, 0.027]
8	668	women	unhealthy	high	middle	0.032[-0.037, 0.103]	0.192 [0.068, 0.317]	0.043 [-0.029, 0.112]	-0.149 [-0.289,-0.018]
9	470	women	unhealthy	high	high	0.720[0.635, 0.805]	0.494 [0.353, 0.636]	0.705 [0.623, 0.790]	0.211 [0.058, 0.366]
10	694	women	unhealthy	low	none	-0.429[-0.498, -0.362]	-0.504 [-0.646, -0.355]	-0.439 [-0.507, -0.370]	0.065 [-0.088, 0.215]
11	741	women	unhealthy	low	middle	-0.285[-0.353, -0.217]	-0.202 [-0.327, -0.079]	-0.282[-0.348, -0.214]	-0.079 [-0.217, 0.054]
12	311	women	unhealthy	low	high	0.049[-0.053, 0.154]	0.099 [-0.045, 0.236]	0.055 [-0.045, 0.151]	-0.044 [-0.197, 0.111]
13	317	men	healthy	high	none	0.100[-0.002, 0.204]	0.027 [-0.111, 0.173]	0.090 [-0.008, 0.189]	0.063 [-0.097, 0.222]
14	525	men	healthy	high	middle	0.258[0.180, 0.337]	0.329 [0.204, 0.450]	0.266 [0.187, 0.345]	-0.062 [-0.2, 0.074]
15	277	men	healthy	high	high	0.531[0.424, 0.641]	0.630 [0.486, 0.772]	0.557 [0.451, 0.662]	-0.073 [-0.237, 0.089]
16	480	men	healthy	low	none	-0.214[-0.298, -0.133]	-0.367 [-0.506, -0.221]	-0.231 [-0.314, -0.150]	0.136 [-0.014, 0.289]
17	493	men	healthy	low	middle	-0.188[-0.271, -0.105]	-0.066 [-0.186, 0.056]	-0.180 [-0.260, -0.100]	-0.114 [-0.25, 0.019]
18	225	men	healthy	low	high	0.134[0.006, 0.253]	0.235 [0.091, 0.372]	0.153 [0.039, 0.268]	-0.082 [-0.241, 0.081]
19	403	men	unhealthy	high	none	-0.240[-0.329, -0.152]	-0.109 [-0.253, 0.041]	-0.231 [-0.319, -0.143]	-0.121 [-0.285, 0.032]
20	508	men	unhealthy	high	middle	0.029[-0.051, 0.110]	0.192 [0.070, 0.320]	0.044 [-0.035, 0.123]	-0.149 [-0.289,-0.013]
21	341	men	unhealthy	high	high	0.796[0.696, 0.896]	0.494 [0.350, 0.636]	0.771 [0.671, 0.871]	0.277 [0.124, 0.441]
22	719	men	unhealthy	low	none	-0.395[-0.463, -0.327]	-0.504 [-0.641, -0.358]	-0.406 [-0.474, -0.340]	0.098 [-0.053, 0.247]
23	711	men	unhealthy	low	middle	-0.212[-0.280, -0.139]	-0.203 [-0.326, -0.081]	-0.213 [-0.280, -0.147]	-0.01 [-0.142, 0.125]
24	331	men	unhealthy	low	high	0.123[0.020, 0.227]	0.099 [-0.045, 0.233]	0.119 [0.026, 0.216]	0.02 [-0.133, 0.179]

*ID* cross-layer number, *N* sample size, *gender* gender, *health* health status, *SES* socioeconomic status, *FP* family participation status, *Predicted sp level* predicted value of social participation level, *Expected sp level* social participation Horizontal expected value, *Random effects* random effect value

lowest,  highest

Therefore, the results support the second research hypothesis that heterogeneity exists in social participation across older adults occupying different social positions. Older women of higher socioeconomic status and in better health have greater family participation, which also promotes their social participation.

## Discussion

Using data from CLASS 2020, the author studied the relationship between family participation and social participation among older adults, as well as the heterogeneity therein. The author found that family participation promotes social participation, and differences exist in social participation across older adults occupying different positions. Specifically, women of higher socioeconomic status and in better health have higher levels of both family participation and social participation, whereas men of lower socioeconomic status and poorer health have the lowest participation. Moreover, socioeconomic status has a greater influence than other variables on differences across intersectional layers, reflecting the cumulative negative impact of other social identity categories on participation. The lack of health resources among older adults is often accompanied by compounding disadvantages stemming from multiple social identities—different social factors reinforce each other, intensifying the social exclusion of disadvantaged older adults.

When considering both aspects of family participation (caregiving and household chores) the author does not find significant conflicts between family and social participation. Hence, participants still have time and energy for social activities [3]. However, future research should further explore the heterogeneity of family participation. While household chores for family members tend to be limited and intermittent, caring for elderly parents or children under the age of three requires more sustained time and energy from older adults. In traditional Chinese culture, blood ties and the continuation of family lineages establish a natural, special intimacy between relatives connected through lineage. Older adults who share housework, such as intergenerational caregiving, embody familial obligations and duties. Thus, family participation represents an important means for older Chinese adults to “remain useful” and find life value after retirement. Research also shows that family participation does not negatively impact psychological health among older Chinese adults. For instance, intergenerational caretaking indirectly improves mental health for middle-aged and older adults by enhancing intergenerational economic support, emotional comfort and social interaction frequency [43]. Additionally, relationships with grandchildren gained through family participation facilitate intergenerational reciprocity from children, expanding older adults’ probability of social interaction [22]. The more diverse the activities older adults engage in, the stronger their motivation to maintain high functional abilities [44]. Family participation promotes older adults’ willingness to engage in social participation. The values, sense of belonging, and social network resources developed through family involvement can encourage older adults to move beyond the family sphere and participate more actively in society.

Regarding the heterogeneity in the relationship between family and social participation, the intersectional analysis results demonstrate unequal patterns of social participation for older adults occupying different positions. This finding shows that these differences are not unidimensional but rather intersectional and complex. Consistent with previous studies, [29–31] the author found that lower socioeconomic status and poorer health among men were associated with lower participation. A higher socioeconomic status and better health among women were associated with greater participation; this group demonstrated the strongest promotion of social participation through family participation. This finding is consistent with older adults’ resource theory, which posits that greater resource endowments facilitate participation [20].

Moreover, according to the intersectional analysis, material resources play the most important role among resources, including finances, health and care. The results show that socioeconomic status has a greater influence on differences across intersectional layers than other variables. However, this requires an appropriate interpretation. This does not necessarily mean that variables such as gender, health and family participation are unimportant. However, socioeconomic status may also reflect the outcomes of socioeconomic and demographic factors that accumulate across the life course. That is, the negative impacts of lower socioeconomic status, poorer health, gender and lack of family participation may accumulate across the life course, eventually resulting in lower participation.

Consistent with previous study, [32] the author also found that groups with higher social participation mostly have higher socioeconomic status, while those at the lower end mostly have lower socioeconomic status. Older adults of higher socioeconomic status possess more abundant social capital and other resources, providing far broader options for social participation [45]. Older adults with sufficient economic resources can engage in preferred social and recreational activities, take up paid jobs instead of excessively demanding volunteer work, or even relocate to communities that better suit their needs [46, 47]. Groups with higher socioeconomic status are also more likely to reside in communities with abundant resources for participation while avoiding adverse environments [48]. Disadvantaged community environments are associated with lower participation, which occurs more often among groups of lower socioeconomic status. One reason is that poorer communities have fewer related resources for participation, including fewer freely available resources, restricting the participation options of these older adults [49]. Hence, the author finds that differences in social participation are closely related to socioeconomic status, demonstrating unequal social participation among older adults.

Additionally, according to the intersectional analysis results, groups lacking health resources were more vulnerable to interaction effects. Intersectionality theory holds that the roots of social inequality lie in mutually reinforcing, tightly intertwined systems of power [50]. No individual social position is equivalent to a simple sum of all identities [51]. Thus, the lack of health resources among older adults is often accompanied by compounding disadvantages stemming from multiple social identities—different social factors reinforce each other. As members of multiple groups, the impacts individuals face regarding participation stem not only from a simple sum of single identity effects (additive effects) but also from multiple synergistic effects of identities on each other (interaction effects) [37]. This intensifies the social exclusion of disadvantaged older adults. Such cross-cutting impacts on participation cannot be attributed to a single factor. Recognizing these intersectional differences in participation barriers is particularly meaningful for policy makers seeking to address participation obstacles faced among less healthy groups.

## Conclusions

Our findings confirm this positive correlation. However, there are several limitations. First, this analysis relied on CLASS 2020 data collected three years ago, so the results may exhibit a time lag. Second, this analysis was cross-sectional and did not consider changes in social strata over time. Moreover, the author could not deeply examine the causal relationships between family and social participation. Moreover, while gender, socioeconomic status, health and family participation are significant correlates of social participation, considering other social dimensions or more detailed identity categories may produce different results. Finally, most of the intersectional MAIHDA research has taken an exploratory approach without prespecified hypotheses for each intersection in the analysis matrix. Nevertheless, this approach provides valuable inductive information on socioeconomic inequalities in older adults’ participation. This novel methodology enhances the understanding of the dynamics underlying privileges and disadvantages that drive participation differences.

The World Health Organization's active aging framework encourages countries to view older adults as valuable resources. In China, this strategy promotes participation, which helps older adults stay active and engaged in life after retirement. the study confirms the positive correlation between family participation and social participation while also identifying groups requiring further efforts to promote participation, particularly older adults with lower socioeconomic status and those in poorer health. These groups require targeted support to increase their participation rates. Policies that address these disparities are crucial to ensuring that all older adults have equal opportunities for engagement in both family and social activities, promoting active aging across different segments of the population.

The Chinese government should prioritize support for these vulnerable groups. First, special attention should be given to older men with low socioeconomic status and poor health. To reduce participation barriers, the government can provide free or low-cost cultural and sports activities within their communities, ensuring access for individuals at all income levels. Second, investment in community resources in low socioeconomic areas should be increased. Expanding and improving public facilities such as sports centers, cultural hubs, and community activity spaces will create more opportunities for social participation. Raising awareness about these resources and ensuring they are easily accessible can help increase participation rates among older adults.

## Data Availability

CLASS data are available through individual user registration. All the details about the application and registration process can be found at https://class.ruc.edu.cn/.

## Appendix

**Table 6:** Linear regression analysis results of family participation and social participation

	(1)	(2)
	social participation level	
Family involvement	0.099***	0.106***
	(0.006)	(0.009)
Gender		0. 055*
		(0.03)
Age		-0.015***
		(0.003)
Married		0.022
		(0.038)
Education level		0.07***
		(0.018)
Hukou		0.067
		(0.044)
Income		0.067***
		(0.012)
Self-rated health		0.006
		(0.018)
Body functions		-0.028***
		(0.005)
Community facilities		0.057***
		(0.01)
Intercept	1.95***	3.13***
	(0.012)	(0.224)
Sample size	10,625	4201
R square	0.037	0.109

Standard errors are in parentheses

^***^*p* < .01, ***p* < .05, **p* < .1
